# Elevated blood pressure among children born to women with obstructed labour in Eastern Uganda: a cohort study

**DOI:** 10.1186/s40885-023-00261-6

**Published:** 2024-02-01

**Authors:** David Mukunya, Milton W. Musaba, Brendah Nambozo, Faith Oguttu, Brian Tonny Makoko, Agnes Napyo, Ritah Nantale, Solomon Wani, Josephine Tumuhamye, Prossy Auma, Ketty Atim, Joan Wamulugwa, Doreck Nahurira, Dedan Okello, Lawrence Ssegawa, Julius Wandabwa, Sarah Kiguli, Martin Chebet

**Affiliations:** 1 Department of Community and Public Health, Busitema University, P.O. Box 1460, Mbale, Uganda; 2 Department of Research, Nikao Medical Center, P.O. Box 10005, Kampala, Uganda; 3 Department of Obstetrics and Gynecology, Busitema University, P.O. Box 1460, Mbale, Uganda; 4 Busitema University Centre of Excellency for Maternal Reproductive and Child Health, Mbale, Uganda; 5 Makerere University Hospital, Makerere University Kampala, Kampala, Uganda; 6 Department of Obstetrics and Gynecology, Mbale Regional Referral Hospital, P.O. Box 921, Mbale, Uganda; 7 Department of Pediatrics and Child Health, Busitema University, P.O. Box 1460, Mbale, Uganda; 8 Department of Research, Sanyu Africa Research Institute, P.O. Box 2190, Mbale, Uganda; 9 Department of Pediatrics, Mbale Regional Referral Hospital, P.O. Box 921, Mbale, Uganda; 10 Department of Pediatrics and Child Health, Makerere University, P.O. Box 7062, Kampala, Uganda

**Keywords:** Elevated blood pressure, Obstructed labour, Uganda, Birth asphyxia, Pediatric hypertension

## Abstract

**Background:**

Globally, high systolic blood pressure accounts for 10.8 million deaths annually. The deaths are disproportionately higher among black people. The reasons for this disparity are poorly understood, but could include a high burden of perinatal insults such as birth asphyxia. Therefore, we aimed to assess the incidence of elevated blood pressure and to explore associated factors among children born to women with obstructed labour.

**Methods:**

We followed up children born to women with obstructed labour aged 25 to 44 months at Mbale regional referral hospital that had participated in the sodium bicarbonate trial ( Trial registration number PACTR201805003364421) between October 2021 and April 2022. Our primary outcome was elevated blood pressure defined as blood pressure (either systolic or diastolic or both) ≥ the 90th percentile for age, height, and sex in the reference population based on the clinical practice guideline for screening and management of high blood pressure in children and adolescents. We used logistic regression to estimate odds ratios between selected exposures and elevated blood pressure.

**Results:**

The incidence of elevated blood pressure was (39/140, 27.9%: 95% (CI: 20.6–36.1)). Participants aged three years and above had twice the odds of elevated blood pressure as those aged less than three years (Adjusted odds ratio (AOR) 2.46: 95% CI (1.01–5.97). Female participants had 2.81 times the odds of elevated blood pressure as their male counterparts (AOR 2.81 95% CI (1.16–6.82). Participants with reduced estimated glomerular filtration rate had 2.85 times the odds of having elevated blood pressure as those with normal estimated glomerular filtration rate (AOR 2.85 95% CI (1.00–8.13). We found no association between arterial cord lactate, stunting, wealth index, exclusive breastfeeding, food diversity and elevated blood pressure.

**Conclusion:**

Our findings show a high incidence of elevated blood pressure among children. We encourage routine checking for elevated blood pressure in the pediatric population particularly those with known risk factors.

## Introduction

Sub-Saharan Africa is experiencing an epidemiological transition from predominantly communicable diseases to a double burden of both communicable and non-communicable diseases [1]. Non-communicable diseases (NCDs) account for 30% of the total burden of disease in sub-Saharan Africa [2]. In 2019, the leading risk factor globally for death was high systolic blood pressure, which accounted for 10·8 million deaths [3]. According to the World Health Organisation, Africa has the highest age-standardised prevalence of hypertension, with 46% of adults older than 25 years being affected [4]. A study conducted among high school children in Uganda found that 11% of the 688 children had elevated blood pressure [5]. Another study in rural Uganda among 1913 children found 44.2% had elevated blood pressure [6].

The onset of hypertension occurs earlier in sub-Saharan Africa compared to high-income countries [1]. The reasons for this are not precisely known, however, a couple of hypotheses exist. Firstly, genetics could have a role to play [7, 8]. Secondly, behaviour, diet and higher burden of known risk factors such as obesity could explain some of the disparity in the burden of hypertension in sub-Saharan Africa [7, 8]. Thirdly, insults such as birth asphyxia that occur in the perinatal period could be partly responsible for this disparity. Birth asphyxia is thought to injure the blood vessels and the heart by causing structural and functional changes and consequently increasing the risk for hypertension [9–11]. In addition, hypertension in children may be related to disease conditions such as kidney disease [9, 10, 12]. Despite the fact that birth asphyxia is one of the most common neonatal conditions in sub-Saharan Africa, there is a paucity of data on the burden of elevated blood pressure among children post-birth asphyxia. Therefore, we aimed to assess the incidence and to explore factors associated with elevated blood pressure among children born to women who experienced obstructed labour in eastern Uganda.

## Materials and methods

### Study design

We conducted a cohort study among children aged between 25 and 44 months, born to women with obstructed labour.

### Study setting

We conducted this study at Mbale Regional Referral and Teaching Hospital (MRRH) between October 2021 and April 2022. MRRH is a public tertiary hospital located in Eastern Uganda with a catchment population of about 4 million people [13]. The hospital serves 16 districts, and is the main referral centre for four district hospitals and 10 health sub-districts. About 12,000 women give birth at MRRH annually, and about 500 of these experience obstructed labour [13].

### Study participants

We recruited children aged 25 to 44 months born to women with obstructed labour. Our participants had previously been enrolled in a clinical trial between July 2018 and September 2019. The parent trial aimed to determine the effect of a sodium bicarbonate infusion on blood lactate and maternal and perinatal outcomes among women with obstructed labour in Mbale Regional Referral Hospital. Participants included in the randomized controlled trial were women with obstructed labour carrying live singleton term pregnancies (≥ 37 weeks of gestation) in cephalic presentation. We excluded those with comorbidities or other obstetric emergencies, such as pre-eclampsia. Trial registration number; PACTR201805003364421 [14].

### Study procedures

The mothers or caregivers of the children were contacted via telephone calls using the contact information obtained at the time of enrollment into the Sodium Bicarbonate trial [15]. We invited the mothers or caregivers to bring their children to Mbale regional referral hospital for clinical assessment. We obtained informed consent from the mothers or caregivers to include their children into the current study. We collected data on the child's medical history, nutrition, growth, and development. A paediatrician or trained medical officer conducted a detailed physical examination.

### Outcome variables

Our outcome variable was elevated blood pressure among children born to women with obstructed labour. Blood pressure was taken using using an automated syphgmomanometer (Model Mindray VS-600, Mindray, Shenzhen, China). The mother or caregiver carried the child on their lap and an appropriate sized paediatric cuff was applied on the left mid upper arm and three measurements were taken. The average of the three systolic and diastolic measurements for each individual were then evaluated for blood pressure for height, age and sex using clinical practice guideline for screening and management of high blood pressure in children and adolescents [16]. We defined elevated blood pressure as a blood pressure value (either systolic or diastolic or both) ≥ the 90th percentile for age, height, and sex in the reference population [16]; but less than the 95th percentile. We defined normal blood pressure as a blood pressure value less than the 90th percentile for age, height, and sex in the reference population [16].

### Independent variables

Our independent variables included the sociodemographic characteristics of the mothers and children, clinical characteristics, nutritional status of the child, wealth index and umbilical arterial lactate at birth. Weight was measured in duplicate to the nearest 100 g using the same electronic scales (Seca model 881 1,021,659, Hamburg, Germany). The length was measured in duplicate to the nearest 1 mm with using a wooden length board. The Stata package “zscore06” was used to calculate weight for height z-score (WHZ) and height-for-age z-score (HAZ). Mid-upper arm circumference (MUAC) was measured in duplicate to the nearest 1 mm, at the midpoint between the olecranon and the acromion process of the left upper limb arm using the samea standard measuring tape. A minimum acceptable diet was defined as the consumption of four of seven (4/7) food groups and at least four [4] meals a day. To get a dietary diversity score, ingredients were categorized into 7 food groups, which include grains, roots and tubers; legumes and nuts; dairy products (milk, yoghurt, cheese); flesh foods (meat, fish, poultry and liver/organ meats); eggs; Vitamin-A rich fruits and vegetables; other fruits and vegetables. Children who satisfied the minimum acceptable diet were coded as 1 while children who didn’t were coded as 0.

### Sample size

The sample size was dependent on the size of the parent study whose aim was to determine the prevalence of neurodevelopmental delay in this cohort [17]. Of the 155 participants enrolled in the parent study, 140 had a blood pressure measurement. This sample size resulted in an absolute precision of 2.3 to 8.2%, i.e., the difference between the point estimate and the 95% confidence interval (CI) for incidence values ranging from 2 to 50%, a precision we deemed adequate.

### Statistical analysis

We entered data using Open Data Kit and then exported it into Stata software Version 14.0 (StataCorp; College Station, TX, USA) for analysis. Continuous variables were summarized into means, medians, standard deviations, and interquartile ranges. We summarized categorical variables into frequencies with percentages. We used logistic regression to estimate odds ratios between selected exposures and elevated blood pressure. This was an exploratory analysis given our small sample size. To calculate blood pressure percentiles in this population; we used a Stata do-file made by Prof Jan Sørensen, Royal College of Surgeons in Ireland, Dublin, Ireland and MD Signe Bruun, Arla Foods Ingredients and Hans Christian Andersen Children's Hospital, Odense University Hospital, Odense, Denmark based on the SAS "%childhoodbppct" macro made by Prof Bernard Rosner, Harvard T.H. Chan School of Public Health, Boston, USA (https://sites.google.com/a/channing.harvard.edu/bernardrosner/pediatric-blood-press/childhood-blood-pressure?authuser=0). The Stata do-files were based on the Clinical Practice Guideline for Screening and Management of High Blood Pressure in Children and Adolescents [16].

## Results

### Study profile

**Fig. 1:**
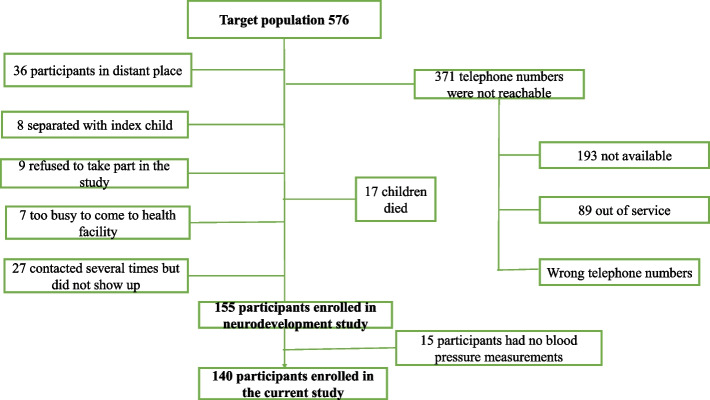
Study profile for children born to women with obstructed labour in eastern Uganda

A total of 576 participants were enrolled in the clinical trial [14]. Only 155 of the 576 came for follow-up [18] and out of the155 only 140 had blood pressure measurements and were included in this current study. Details are shown in Fig. 1.

### Participant characteristics

We recruited 140 participants whose mean age was 25.5 months (standard deviation of 6.3 months), and the age range was between 25 to 44 months. More than half of the participants were below three years (90/140, 64.3%). Majority of the participants were male (94/140, 67.1%). Slightly less than half (64/140,46.4%) had arterial lactate of 4.8-10 mmol/L at birth and less than two thirds had exclusively breatfed (84/140, 60%). Majority (125/140, 89.3%) were HIV negative. Details are shown in Table 1.

**Table 1:** Socio-demographic characteristics of children born to women with obstructed labour

Factor	Normal blood pressure (< 90th percentile) *n* = 101(73%)	Elevated blood pressure (> = 90th Percentile) *n* = 39(27%)	Total *N* = 140
**Age**	Mean = 25.7(6.3)	Mean 25.1(6.2)	Mean 25.5 (6.3)
**Age of child**			
Below 3 years	68(67.3)	22(56.4)	90(64.3)
3 years and above	33(32.7)	17(43.6)	50(35.7)
**Sex**			
Male	74(73.3)	20(51.3)	94(67.1)
Female	27(26.7)	19(48.7)	46(32.9)
**Father’s educational level**			
Primary	31(30.7)	11(28.2)	42(30)
Secondary	46(45.5)	16(41)	62(44.3)
Tertiary	24(23.8)	12(30.8)	36(25.7)
**Wealth index**			
Q1 (Poorest)	20(19.8)	8(20.5)	28(20)
Q2	27(26.7)	3(7.7)	30(21.4)
Q3	15(14.9)	14(35.9)	29(20.7)
Q4	22(21.8)	6(15.4)	28(20)
Q5 (Richest)	17(16.8)	8(20.5)	25(17.9)
**Child’s birth order**			
Firstborn	46(45.5)	21(53.8)	67(47.9)
2–4	37(36.6)	14(35.9)	51(36.4)
5–12	18(17.8)	4(10.3)	22(15.7)
**Arterial lactate**			
min-4.8	20(20.2)	11(28.2)	31(22.5)
4.8–10	46(46.5)	18(46.2)	64(46.4)
10-max	33(33.3)	10(25.6)	43(31.2)
**Admission to Neonatal unit**			
Yes	16(15.8)	6(15.4)	22(15.7)
No	85(84.2)	33(84.6)	118(84.3)
**Admission for any illness in the last one year**			
Yes	14(13.9)	9(23.1)	23(16.4)
No	87(86.1)	30(76.9)	117(83.6)
**Oxygen afterbirth**			
No	91(90.1)	36(92.3)	127(90.7)
Yes	10(9.9)	3(7.7)	13(9.3)
**Neonatal fever**			
No	84(83.2)	36(92.3)	120(85.7)
Yes	17(16.8)	3(7.7)	20(14.3)
**Hospital admission for severe malaria in last one year**			
No	89(88.1)	31(79.5)	120(85.7)
Yes	12(11.9)	8(20.5)	20(14.3)
**Ever convulsed**			
No	93(92.1)	34(87.2)	127(90.7)
Yes	8(7.9)	5(12.8)	13(9.3)
**HIV status**			
Negative	89(88.1)	36(92.3)	125(89.3)
Positive	12(11.9)	3(7.7)	15(10.7)
**Early breastfeeding initiation**			
Not done	39(38.6)	16(41)	55(39.3)
Done	62(61.4)	23(59)	85(60.7)
**Food diversity**			
No	9(8.9)	8(20.5)	17(12.1)
Yes	92(91.1)	31(79.5)	123(87.9)
**Stunting**			
No	81(80.2)	32(82.1)	113(80.7)
Yes	20(19.8)	7(17.9)	27(19.3)
**Wasting**			
No	99(98)	36(92.3)	135(96.4)
Yes	2(2)	3(7.7)	5(3.6)
**Minimum Number of meals**			
< 4 meals a day	32(31.7)	12(30.8)	44(31.4)
4 and above meals	69(68.3)	27(69.2)	96(68.6)
**Exclusive breastfeeding**			
Yes	58(57.4)	26(66.7)	84(60)
No	43(42.6)	13(33.3)	56(40)

### Incidence of elevated blood pressure among children born to women with obstructed labour

The incidence of elevated blood pressure was 27% (39/140). The total follow-up period for the study participants was 407.42 years and the incidence rate was 9.57 per 100 person years.

### Factors associated with elevated blood pressure among children with obstructed labour

Participants aged three years and above had twice the odds of elevated blood pressure as those aged less than three years (adjusted odds ratio [AOR] 2.46 95%CI (1.01–5.97). Female participants had 2.81 times the odds of elevated blood pressure as males (AOR 2.8195% CI (1.16—6.82). Participants with severe malaria in the last year had 3.6 times the odds of elevated blood pressure as their counterparts (AOR 3.6 95% CI (1.09 -11.88). Participants with reduced estimated glomerular filtration rate had 2.85 times the odds of elevated blood pressure as those with normal estimated glomerular filtration rate (AOR 2.85 95% CI (1.00–8.13). No association was found between umbilical arterial lactate, stunting, wealth index, exclusive breastfeeding, food diversity and elevated blood pressure (Table 2).

**Table 2:** Multivariable analysis for factors associated with elevated blood pressure and hypertension among children born to women with obstructed

Factors *N* = 140	COR 95% CI	*p*-value	AOR 95% CI	*P* value
**Age**				
Less than 3 years	1		1	
3 years and above	1.59(0.75–3.40)	0.229	2.56(1.03–6.35)	0.043
**Sex**				
Male	1		1	
Female	2.6(1.21–5.61)	0.014	3.2(1.27–8.05)	0.013
**Arterial lactate level**				
< 4.8	1		1	
4.8 – 10	0.71(0.28–1.78)	0.466	0.66(0.22–1.92)	0.441
> 10	0.55(0.20–1.53)	0.252	0.57(0.17–1.91)	0.361
**Stunting**				
No	1		1	
Yes	0.89(0.34–2.30)	0.803	1.58(0.52–4.79)	0.418
**Wealth index**				
Upper-income status	1		1	
Middle-income status	2.6(1.01–6.73)	0.049	2.29(0.76–6.90)	0.141
Lower-income status	0.65(0.27–1.60)	0.35	0.44(0.16–1.24)	0.121
**Glomerular filtration rate**				
Normal estimated glomerular filtration rate	1		1	
Reduced estimated glomerular filtration rate	1.85(0.79–4.31)	0.157	2.85(1.00–8.13)	0.05
**Exclusive breastfeeding**				
No	1		1	
Yes	0.67(0.31–1.46)	0.318	0.51(0.20–1.30)	0.159
**Hospitalization for severe malaria in the past year**				
No	1		1	
Yes	1.91(0.72–5.12)	0.196	3.94(1.15–13.58)	0.03
**Food diversity**				
No	1		1	
Yes	0.38(0.13–1.07)	0.066	0.4(0.12–1.37)	0.144

*COR* Crude odds ratio, *AOR* Adjusted odds ratio

## Discussion

In this cohort of children born to women with obstructed labour at Mbale Hospital, the incidence of elevated blood pressure was high (27%) with an incidence rate of 9.57 per 100 person years. The factors associated with elevated blood pressure were age, sex of the child and a reduced estimated glomerular filtration rate.

### Incidence of elevated blood pressure

Our study found a high incidence of elevated blood pressure. This is contrary to findings of Noubiap et al*.* [19], Afaa et al*.* [20] and Ejike et al*. *[21]*,* who found a lower incidence of elevated blood pressure**.** This difference could be attributed to variations in the study populations. Unlike the other studies, our study population was a cohort of children born to women with obstructed labour. These children were exposed to intrapartum hypoxia and probably asphyxia and acidosis [22]. As a result, these children might have suffered some level of persistent chronic multi-organ dysfunction. Our findings could imply that many children might be having elevated blood pressure or childhood hypertension but remain undiagnosed because blood pressure measurements are not routine in our setting. Our study suggests the need to routinely measure blood pressure for children following birth asphyxia. This is important for early identification and timely management of children with elevated blood pressure.

### Factors associated with elevated blood pressure among children

Our study found an association between age and elevated pressure. Participants aged three years and above had twice the odds of elevated blood pressure as those aged less than three years. This is consistent with findings from other studies which found an association between an increase in age and elevated blood pressure among children [23–27]. An age-related increase in blood pressure could be attributed to the changes in arterial and arteriolar stiffness that occur with increase in age [28–30]. However, our finding differs from those of Chiolero et al., who found a higher elevated blood pressure among younger children compared to older children [31].

The female participants had twice the odds of elevated blood pressure as their male counterparts. Our finding is similar to those from other studies who also reported an association between the female gender and elevated blood pressure among children [23, 32–35]. However, our findings were inconsistent with majority of the literature that suggests males are more likely to have elevated blood pressure [36–38]. This disparity maybe explained by the fact that females are more likely to experience the white coat effect than the males [20, 39, 40]. The white coat effect is an anxiety induced elevation in blood pressure in the presence of a health worker or a health facility. However, our findings differ from those of Muhihi et al*.*, Mushengezi et al., and Chillo et al., and a recent systematic review did not find an association between gender and elevated blood pressure in children [19, 24, 41]. We recommend measurement of ambulatory blood pressure to rule out this effect.

Children with reduced estimated glomerular filtration rate had twice the odds of elevated blood pressure as those with normal estimated glomerular filtration rate. This could be explained by the fact that this cohort was born to women with obstructed labour and could have suffered perinatal asphyxia which compromised renal perfusion as blood is shunted to the brain and heart [42]. This could have resulted in hypoxic tissue injury and subsequent irreversible kidney damage [43–45]. As a result, there are alterations in fluid and electrolyte balance which is associated with elevated blood pressure in children [46].

Our study did not find an association between exclusive breastfeeding and blood pressure. However, some literature suggests that breastfeeding is protective against elevated blood pressure in childhood [47–49]. In addition, our study found no association between arterial lactate with elevated blood pressure, stunting, and wealth index.

### Strength and limitations

To the best of our knowledge, our study is among the first to assess elevated blood pressure among children born to women diagnosed with obstructed labour, a major cause of neonatal morbidity and mortality in low-resource settings. The main weakness in our study was a low response rate, which makes our study prone to selection bias. The direction of bias could have been either way as those who did not come for follow-up could have been healthier or sicker.

## Conclusion

Our findings show a high incidence of elevated blood pressure among children. This implies that there is an urgent need to investigate the other risk factors of elevated blood pressure in this category. This is important since there is evidence of a link between elevated blood pressure in childhood and hypertension in adulthood. We encourage routine checking for elevated blood pressure in the pediatric population particularly those with known risk factors. We also encourage further research on elevated blood pressure among the pediatric population ins sub-Saharan Africa.

## Data Availability

The datasets used and/or analyzed during the current study are available from the first author through reasonable request.
